# Zero-Contrast Transcatheter Aortic Valve Implantation vs. Standard Practice: Periprocedural and Long-Term Clinical Outcomes

**DOI:** 10.3390/jcm13185405

**Published:** 2024-09-12

**Authors:** Roberto Nerla, Elisa Mikus, Angela Sanseviero, Angelo Squeri, Simone Calvi, Carlo Savini, Diego Sangiorgi, Fausto Castriota

**Affiliations:** 1 Interventional Cardiology Unit, GVM (Gruppo Villa Maria) Care & Research, Maria Cecilia Hospital, 48033 Cotignola, Italy; robertonerla83@gmail.com (R.N.); angelasanseviero192@gmail.com (A.S.); asqueri@gvmnet.it (A.S.); fcastriota@gvmnet.it (F.C.); 2 Cardiac Surgery Unit, GVM Care & Research, Maria Cecilia Hospital, 48033 Cotignola, Italy; scalvi@gvmnet.it (S.C.); csavini@gvmnet.it (C.S.); dsangiorgi@gvmnet.it (D.S.); 3 Department of Experimental Diagnostic and Surgical Medicine (DIMEC), University of Bologna, 40133 Bologna, Italy

**Keywords:** transcatheter aortic valve implantation, acute renal injury, no contrast

## Abstract

**Background**: We aimed to compare the procedural efficacy and long-term clinical results of a totally contrast-free Transcatheter Aortic Valve Implantation (TAVI) procedure (i.e., contrast dye was not used for either the pre-procedural assessment or during the procedure) to those of standard practice in patients with severe renal dysfunction. **Methods**: All consecutive patients with a glomerular filtration rate (GFR) ≤ 35 mL/min and severe aortic stenosis who were treated with transfemoral TAVI at our Institution were included in the registry. The zero-contrast patients underwent carbon dioxide angiography and a non-contrast CT scan for assessment of vascular access suitability, and aortic annulus sizing was performed by a TEE, and the procedural guidance was fluoroscopic and echocardiographic. Procedural outcomes were evaluated, and clinical long-term follow-up was performed for all included patients. **Results**: A total of 44 patients (median age, 85 (IQR, 80.75–87.00)) were included in the zero-contrast group (TEE guidance and general anesthesia in 37 (84%) patients), while 63 patients were included in the standard practice arm (82 ± 78 mL of contrast dye used). Procedural success was obtained in 100% of cases. There were no differences in procedural outcomes, including final mean aortic gradients (5.5 (IQR, 5.0–10.0) mmHg in the zero-contrast group vs. 6.0 (IQR, 5.0–10.0) mmHg in the standard practice group) and rate of at least a moderate paravalvular leak (0% vs. 1.6% in the zero-contrast and standard practice groups, respectively; *p* = 0.31). No differences in AKI during the hospital stay were observed. Over a median follow-up of 3.3 years, there was a significantly lower rate of AKI (1.2% vs. 25.9%, *p* < 0.001) and rehospitalizations (1.6% vs. 35.5%, *p* < 0.00) in standard practice group. **Conclusions**: We showed for the first time the feasibility and efficacy of a totally contrast-free strategy compared to standard practice in TAVI patients with severe renal dysfunction. Besides achieving comparable procedural results, the zero-contrast strategy showed a better long-term clinical outcome in reducing hospital readmissions for kidney function deterioration.

## 1. Introduction

Chronic kidney disease is one of the most frequent comorbidities in TAVI patients, with a prevalence ranging from 5.6% to up to 56.3% [1,2,3,4,5,6]. Of note, severe renal dysfunction is the most powerful predictor of peri-procedural acute kidney injury (AKI), which itself is associated with increased morbidity and mortality after TAVI [7,8,9,10]. Despite being related to multiple possible causes (i.e., anemia, hypotension, heart failure, age, diabetes, etc.), peri-procedural AKI following TAVI is usually related to contrast-induced nephropathy. Contrast-induced nephropathy is considered an acute decrease in renal function following administration of iodinated contrast media, which usually normalizes in 1–3 weeks [11]. Different strategies have been suggested to prevent renal damage in high-risk patients undergoing TAVI [12,13]. Among all the avoidable causes of contrast-induced nephropathy, the total amount of administered contrast dye before (procedural planning) and during TAVI plays a crucial role [14,15]. Of note, the volume of contrast to creatinine ratio was found to predict early mortality and AKI after TAVI [16]. As a consequence, a number of measures aimed at reducing the amount of contrast media used during TAVI have been proposed to reduce the incidence of kidney injury in high-risk patients [17,18].

We previously showed for the first time the feasibility of a totally contrast-free TAVI, including both pre-procedural evaluation and procedural guidance [19]. However, no data is available regarding the outcomes of such a strategy compared to standard practice in patients with severe renal dysfunction undergoing TAVI.

The aim of this study was to assess safety and efficacy of this standardized ‘zero-contrast’ approach compared to the standard clinical practice that is used to treat patients with severe renal dysfunction undergoing TAVI.

## 2. Materials and Methods

All consecutive patients with severe renal dysfunction (i.e., GFR ≤ 35 mL/min) treated by TAVI at our Institution between 2017 and 2023 were prospectively included in a clinical database, which was regularly checked for data completeness. Clinical follow-up was performed as part of the retrospective TAVI registry of the Institution.

Allocation to the zero-contrast arm was related to the operator performing the procedure, due to the fact that only some operators performing TAVI during the time frame for the study had experience with non-contrast strategies.

Since our center is a tertiary one for structural interventions, initial patient screening was performed at spoke centers and included a conventional transthoracic echocardiogram to confirm the presence of severe aortic stenosis and a coronary angiogram to rule out coronary artery disease. After the initial evaluation, patients were addressed to TAVI after a local multidisciplinary Heart Team meeting that was usually held at least one month after the procedure.

The use of an alternative access (i.e., transaortic or transapical) was considered an exclusion criterion for the study.

### 2.1. Zero-Contrast Arm

**Figure 1:**
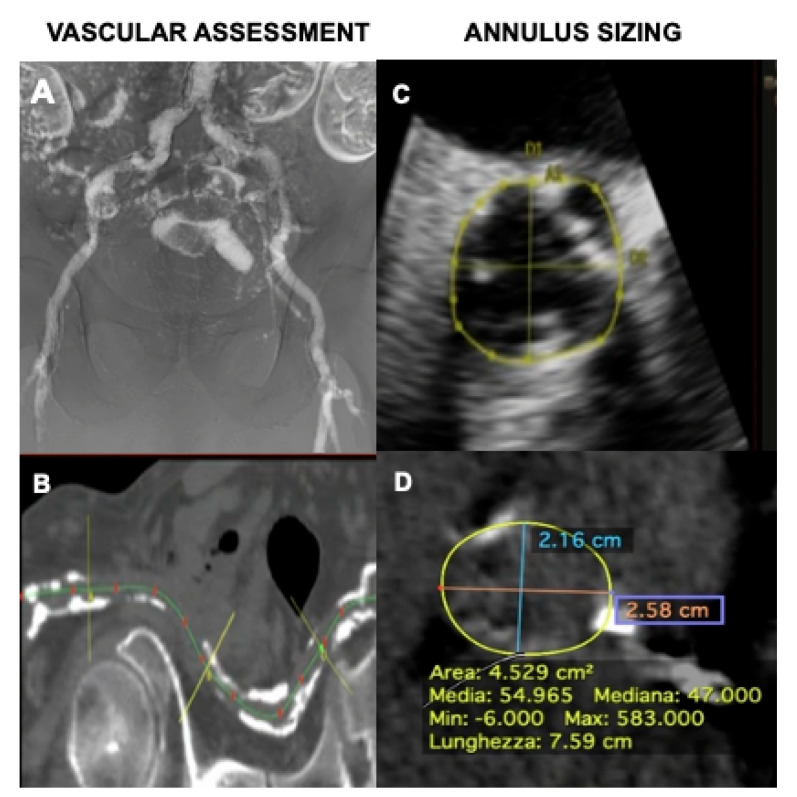
Zero-contrast strategy. The evaluation of vascular anatomy in the zero-contrast group was performed by CO_2_ angiography (panel **A**) to assess patency and diameter of the vessels and non-contrast CT to depict the amount, extension, and localization of vascular calcifications (panel **B**). Annulus sizing was performed by merging information from 3D TEE aortic annulus measurements (panel **C**) and non-contrast CT reconstruction (panel **D**).

The zero-contrast approach is summarized in Figure 1. As a part of routine pre-procedural assessment, non-contrast CT was performed to localize and quantify the degree of aortic calcification as well as the amount and distribution of calcium in iliac and femoral arteries. To evaluate the suitability of the iliofemoral axis for the planned device to be implanted, CO_2_ angiography was performed in all patients the day before the procedure. CO_2_ angiography is currently used as a contrast agent for aortography, as well as for outflow assessment, renal arteriography, and visceral angiography, and to guide lower limb interventions, since its use is not associated with nephrotoxicity or allergic reactions. CO_2_ is approximately 400 times less viscous than iodinated contrast medium. The low viscosity permits even manual gas injection with small-bore catheters; however, being less dense than iodinated contrast medium, it requires digital subtraction angiography with good contrast resolution for gas imaging. Among CO_2_ advantages, the absence of allergic reactions and renal toxicity make this the currently preferred technique for renal artery angioplasty and stent placement [20]

A transesophageal echocardiogram (TEE) was used for annulus sizing. As reported, aortic annular measurements obtained from 3D echocardiography are accurate and reproducible [21]. The aortic valve and the ascending aorta were studied in short-axis (midesophageal, 40°–70°) and long-axis views (midesophageal, 110°–135°), and 2D images with and without color Doppler were obtained and stored in the hospital cardiology imaging archive (Estensa Suite). Using the “3D zoom” feature, 3D data sets of the aortic valve were acquired. Maximum and minimum annulus diameters and valve area were reported for device sizing. However, useful information was derived from preliminary non-contrast CT performed before admission to identify valve calcifications and measure ascending aorta size (Figure 1).

Valve final sizing was determined as per synthesis of all the available information, including transthoracic and/or TEE, non-contrast CT, and calcification on fluoroscopy.

As a pre-specified protocol, patients were treated for aortic stenosis with zero-contrast TAVI at least one month after having undergone contrast dye administration during a coronary angiogram.

Procedural guidance was fluoroscopic for implantation views, with confirmation of the best 3-cusp and COT views using the “3-pigtails” technique [19]. The assessment of implantation depth was made with the TEE long-axis view in general anesthesia. However, in the presence of a good transthoracic window, the TTE was used for procedural guidance in a small subset of patients.

All valve types and sizes were included in the protocol. Balloon pre-dilatation was performed at the discretion of the operator before valve deployment with non-contrast injection. Final aortic regurgitation was assessed by echocardiography.

### 2.2. Standard Practice Arm

Patients with severe renal dysfunction treated by operators with no experience with the zero-contrast technique were included in the study. Institutional guidelines for standard practice to perform TAVI in this setting included the use of a maximum amount of 60 mL for angio-CT (including aortic root and peripheral vascular assessment).

### 2.3. Procedural and Clinical Outcome Assessment

Main procedural and clinical outcomes were defined according to VARC-3 criteria [22]. As a standard procedure, serum creatinine was usually checked in all TAVI patients every 24 h during the 3 days after the procedure and every time there was at least a 25% increase compared to basal value. In addition, a final creatinine check was performed at discharge.

### 2.4. Statistical Analysis

After checking normal distribution with a Shapiro–Wilk test, continuous variables were reported as mean and standard deviation (SD) or median and 1st–3rd quartile (IQR) and compared with a Student’s *t*-test or Mann–Whitney test; categorical variables were reported as absolute number and frequencies and compared with a Fisher’s exact test. To balance the groups, the inverse probability of treatment weighting (IPTW) with covariate balancing propensity score (CBPS) method was applied. The IPTW is a method used in observational studies to adjust for confounding. In the IPTW procedure, each patient in the study is assigned a weight, as estimated by the propensity score. Applying these weights to the study population creates a pseudo-population where confounders are equally distributed between groups, thereby balancing baseline characteristics and reducing bias in the analysis. Gender, age, BMI, atrial fibrillation, family history of CAD, hypertension, current smoking, dyslipidemia, previous MI, previous CABG, CAD, PAD, previous CVA, COPD, diabetes mellitus, hemoglobin, creatinine, eGFR, logistic_Euroscore, and EF were used for weights estimation. No missing data were present. The Absolute standardized mean differences (ASMD) were reported in order to assess balancing across groups; variables with ASMD < 0.2 were considered as balanced; weighted means (SD) and percentages were reported after IPW. Weighted univariate Cox proportional hazard regression models were reported for outcomes; weights were derived from the IPTW. Weighted Kaplan–Meier and associated weighted log-rank tests were also produced. Multivariable Cox regression with LASSO (Least Absolute Shrinkage and Selection Operator) selection with 50-fold cross-validation (LOOCV) for variable selection was assessed to identify predictors of death on the overall sample; the same set of covariates presented above was used. All analysis were performed with R 4.4.0 (R Foundation for Statistical Computing, Vienna, Austria); *p*-values < 0.05 were considered statistically significant.

## 3. Results

From December 2017 to December 2023 a total number of 107 patients were included in the study. The procedure was successfully performed via transfemoral access in all patients.

A total of 44 patients (median age 85 (IQ 80.75–87.00)) were included in the zero-contrast population. In 37 patients, a TEE was used to guide the procedure under general anesthesia. In the remaining 7 patients, mild sedation was used, and a transthoracic echocardiogram (TTE) successfully guided the procedure. CO_2_ angiographic pictures were interpretable in 100% of cases. In two cases, vessel preparation with intravascular lithotripsy for severe calcification was performed before advancing the delivery system.

On the other hand, 63 patients were included in the standard practice arm. In this group, a mean amount of 82 ± 78 mL contrast dye was used to undertake the procedure.

The main clinical characteristics of enrolled patients are reported in Table 1. Of note, patients’ global risk was very high in both groups, with a significant general clinical frailty reported for all cases. Patients included in the zero-contrast group tended to have worse renal function before IPW correction.

Baseline echocardiographic evaluation showed no significant differences between the two groups with regards to ejection fraction, concomitant mitral valve disease and severity of aortic stenosis (Table 2).

Procedural details, including the need for pre-dilatation and post-dilatation, are summarized in Table 3. All TAVI devices available at our laboratory were used according to a strategy tailored to aortic root anatomy and patient’s characteristics. As a choice, only self-expandable valves were chosen, in order to potentially counterbalance a sizing mistake in annulus evaluation.

### 3.1. Procedural Outcomes

**Figure 2:**
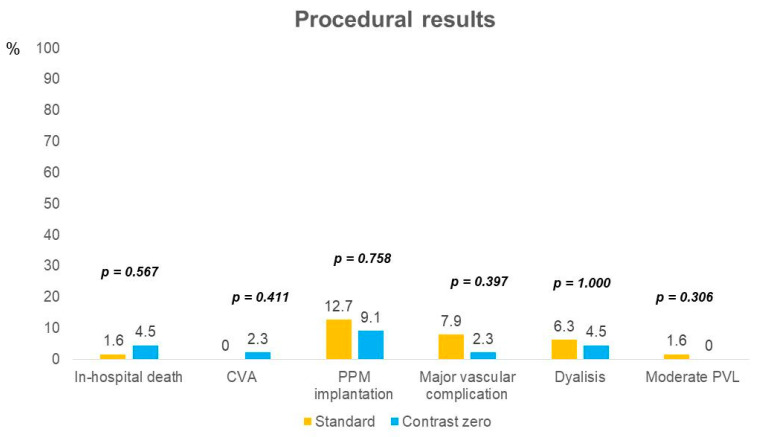
Procedural and clinical in-hospital outcomes. Comparison of clinical events and procedural results between the two groups of the study.

Procedural results are shown in Figure 2. There were no significant differences in acute procedural and clinical outcomes between the two groups. The mean aortic gradient at discharge was 5.5 (IQ 5.0–10.0) mmHg in the zero-contrast group vs. 6.0 (IQ 5.0–10.0) mmHg in the standard practice group (*p* = 0.67).

In the zero-contrast group, procedural success was obtained in 100% of patients. All patients had no more than mild aortic regurgitation. Two patients died during their hospital stay: one had a gastrointestinal bleeding in the context of a known hepatic disease with spontaneously raised INR on day 2 after TAVI and developed multi-organ failure; the second one developed a progressive reduction of respiratory function with bilateral pleural effusions requiring intubation on day 3 after TAVI. In both groups, renal function deteriorated after worsening of general clinical conditions.

There was one major vascular complication consisting of a significant hemoglobin drop in a patient with known history of anemia, thus requiring transfusion of two RBC units. One patient developed acute pericardial effusion during the procedure due to wire perforation during valve-crossing into a small hypertrophic left ventricle outflow tract; emergent pericardiocentesis was performed immediately and the procedure was carried out without the use of any contrast dye.

Excluding the two aforementioned patients requiring ineffective dialysis, no other patients developed acute kidney injury at 48 h or at discharge.

### 3.2. Long-Term Clinical Outcomes

The follow-up duration was comparable between the two groups (zero-contrast group median, 44.26 (IQR, 9.34–59.11) vs. standard practice group median, 38.30 (IQR 14.26–50.92); *p* = 0.52).

**Figure 3:**
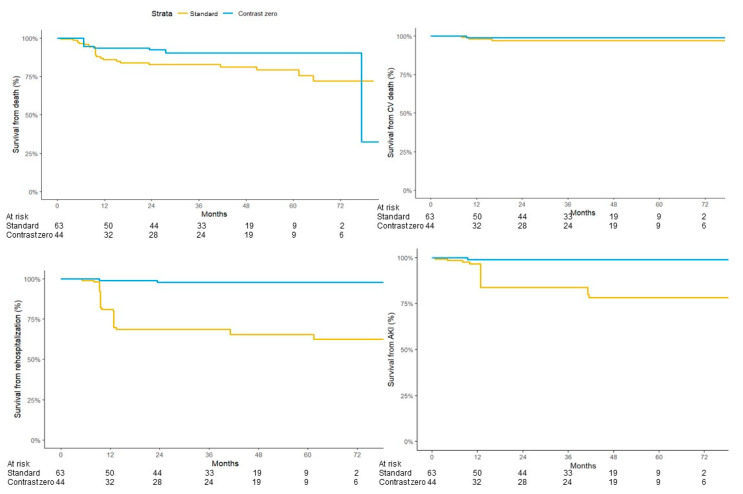
Kaplan–Meyer curves for long-term clinical events. Kaplan–Meyer curves depicting Cox regression analysis for survival from death (top left, *p* = n.s.), survival from CV death (top right, *p* = n.s.), survival from rehospitalization (bottom left, *p* < 0.001) and survival from AKI (bottom right, *p* = 0.01).

Clinical events registered at follow-up are summarized in Table 4. While only a non-significant trend towards a lower any-cause mortality associated with zero-contrast strategy may be suggested, there was a statistically significant lower number of rehospitalizations for any cause and a statistically significant lower number of AKI in the contrast zero-group (Figure 3).

The Cox proportional hazard model with LASSO selection for the risk of death showed that only left ventricular ejection fraction was able to predict the outcome during follow-up (HR, 0.93, (95% CI, 0.89–0.97); *p* = 0.001).

## 4. Discussion

Chronic kidney disease is a relevant and increasing worldwide problem with known prognostic implications. A meta-analysis including nine studies with a total of 4992 patients exploring the impact of pre-existing kidney disease on TAVI outcomes showed that both moderate and severe renal impairment were associated with adverse prognosis [23]. The reported rates of AKI after TAVI are usually related to patients’ risk profiles and comorbidities [24]. In any way, AKI is associated not only with an almost three-fold increased risk of developing chronic kidney disease but also with up to a four-fold higher mortality rate [25]. In the particular setting of TAVI, patients might develop kidney injury after the procedure for several factors, including contrast agent administration, concomitant drugs, need for rapid pacing with resulting hypotension, renal hypoperfusion, blood loss, embolization during the implantation due to patient’s age, frequent coexistence of atherosclerosis, or postoperative severe inflammatory response syndrome [26].

Although other factors might contribute to renal damage, contrast load is still among the most relevant ones. The recently published SWEDEHEART registry showed that patients developing AKI were those who had received a higher contrast volume [27]. In addition, there is a need for adequate procedural planning to assess annulus measurements and peripheral vessel suitability for the procedure, and there is a responsibility to account for any additional and usually not detectable risk of renal deterioration days or weeks before the TAVI procedure. Therefore, a tailored strategy that consists of using no contrast dye at all, both in the planning and in the procedural phases, would be strongly desirable.

Preventative strategies in this setting have traditionally consisted of the administration of intravenous fluids, including sodium chloride and sodium bicarbonate, before the administration of contrast medium [28] and the use of a RenalGuard system performing real-time automated hydration balance using a closed-loop isotonic volume hydration monitoring and infusion system [29]. Nonetheless, recent literature suggested that sodium-glucose cotransporter-2 inhibitors, despite an initial alarm, may exert favorable effects on AKI in patients without diabetes mellitus [30]. Unfortunately, none of our patients was under these medications.

Besides preventative measures, strong advice to reduce contrast dye in high-risk groups is part of routine recommendation. In this setting, our study showed, for the first time, that a full contrast-free TAVI treatment in patients with advanced renal failure is able to achieve, at least, non-inferior procedural and clinical in-hospital outcomes than those obtained with standard practice. In our population, through assessment of vascular suitability for the intervention by combining non-contrast CT data and CO_2_ angiography (not previously described for this specific setting), we were able to achieve successful valve deployment in all cases, with no relevant vascular complications. Similarly, the use of a 3D measurement of annulus size, although theoretically having been associated with the risk of downsizing the device, has been confirmed to be safe and reliable in the choice of the valve [31], with no evidence of paravalvular leaks at hospital discharge. The use of different valve platforms, in our experience, confirmed the suitability of the technique for all valves, with no difference in outcomes derivable from this small population. However, the self-expanding nature of the devices and the repositionability of most of them should be considered as paramount features in order to avoid any possible mistake in valve sizing or positioning.

Besides providing evidence of the non-inferiority of the zero-contrast approach to standard practice, our data suggest a better long-term outcome associated with contrast-sparing techniques. An increasing number of reports are showing the advantages of avoiding contrast dye not only during TAVI procedure but also before the TAVI procedure [32,33]. However, being isolated reports with low numbers and no control group, they do not provide real indication about the feasibility of a standardized zero-contrast strategy. Instead, our data suggest a possible clinical benefit coming out after a longer than expected follow-up, whereas no clear advantage, in terms of AKI incidence, was observed during index hospitalization. Our patients showed a lower number of AKI and rehospitalization during midterm follow-up, possibly suggesting that sparing contrast might exert a long-term protection in patients with frail kidney function. This is in keeping with previous observations suggesting that worsened renal function can occur at a later stage after TAVI and is associated with increased mortality [34].

While the clinical benefit of the zero-contrast strategy was evident over time, there was no statistically significant reduction of AKI during index hospitalization. This is not surprising when considering other reports suggesting that the impact of contrast administration on kidney function in TAVI patients with normal kidney function may be better tolerated because of the hemodynamic changes following aortic valve replacement [35]. In addition, the use of general anesthesia in most zero-contrast patients, although well tolerated, could have mitigated the relevance of the hemodynamic improvement after valve replacement in this group of patients, thus explaining this negative result.

Taken together, our results confirm the feasibility of a zero-contrast approach with the majority of currently available TAVI devices and show non-inferior procedural results compared to standard practice. Given the suggested clinical benefit over long-term follow-up of this strategy and the lack of clear indications for high-risk groups, its standardization may have relevant clinical benefits. Moving TAVI to all comers, it will be of paramount importance to identify high-risk patients (i.e., those with pre-existing renal dysfunction, diabetes mellitus, and/or cognitive impairment) and to promote optimizing strategies to improve outcomes, including: (1) volume optimization before the procedure, reduction of paravalvular regurgitation, bleeding, and vascular complications; (2) restriction of nephrotoxic drugs, mainly contrast agents; (3) prevention of hypoglycemia; and (4) total avoidance of a contrast dye. The opportunity to extend these recommendations to larger populations should be built upon properly sized randomized trials in high-risk subgroups.

### Limitations of the Study

Several limitations should be noted. First, the low number of patients and the non-randomized nature of the study makes it impossible to drive definite conclusions regarding the efficacy of this approach compared to the standard one. Second, the studied population represents a very high-risk setting in whom the weight of multiple comorbidities could make difficult to understand (at least in patients with clinical events) the relevance of renal function oscillations after TAVI procedure. Third, the impact of general anesthesia in such a high-risk subset of patients could be not neglected.

## 5. Conclusions

Our results showed for the first time the feasibility and the efficacy, compared to standard practice, of a totally contrast-free strategy for performing TAVI in patients with poor renal function. Zero-contrast TAVI, including planning and procedural standardized steps, should be considered a valuable option to be properly investigated in adequately sized prospective clinical trials. Of note, future studies will need to clarify the possible extension of these benefits to patients with moderate renal dysfunction in whom residual kidney function may account for larger improvements over time. Furthermore, molecular changes in kidney cells after TAVI that may be related to using or not any contrast dye have never been investigated and may suggest interesting pathophysiological experimental studies in this challenging scenario.

## Figures and Tables

**Table 1:** Baseline clinical characteristics in standard practice vs. the zero-contrast arm before and after IPW.

	Standard (*n* = 63)	Zero-Contrast (*n* = 44)	*p*	SMD	Standard (*n* = 63)	Zero-Contrast (*n* = 44)	Weighted SMD
Male sex, *n* (%)	28 (44.4)	20 (45.5)	1.000	0.020	46.2	44.6	0.033
Age, median (IQR)	85.00 (80.50, 87.00)	85.00 (80.75, 87.00)	0.884	0.081	83.2 (5.2)	83.6 (5.5)	0.064
BMI, mean (SD)	24.03 (3.73)	26.85 (3.94)	<0.001	0.736	25.8 (4.6)	26.2 (3.5)	0.084
Atrial fibrillation, *n* (%)	18 (28.6)	21 (47.7)	0.066	0.402	33.5	33.2	0.005
Family history of CAD, *n* (%)	4 (6.3)	3 (6.8)	1.000	0.019	12.1	13.4	0.052
Hypertension, *n* (%)	57 (90.5)	44 (100.0)	0.035	0.507	95.5	100.0	0.217
Current smoking, *n* (%)	2 (3.2)	0 (0.0)	0.511	0.256	17.5	16.3	0.030
Dyslipidemia, *n* (%)	42 (66.7)	35 (79.5)	0.190	0.294	75.7	79.2	0.081
Previous MI, *n* (%)	13 (20.6)	7 (15.9)	0.620	0.123	30.5	31.8	0.034
Previous CABG, *n* (%)	12 (19.0)	8 (18.2)	1.000	0.022	17.3	17.4	0.001
CAD, *n* (%)	38 (60.3)	24 (54.6)	0.558	0.116	62.6	62.5	0.001
PAD, *n* (%)	12 (19.0)	12 (27.3)	0.352	0.196	18.7	19.8	0.025
Previous CVA, *n* (%)	12 (19.0)	3 (6.8)	0.093	0.371	9.1	2.7	0.194
COPD, *n* (%)	14 (22.2)	14 (31.8)	0.275	0.217	27.2	30.1	0.066
Diabetes mellitus, *n* (%)	16 (25.4)	17 (38.6)	0.202	0.287	30.9	32.2	0.027
Hemoglobin (g/dL), mean (SD)	11.58 (1.56)	11.22 (1.63)	0.263	0.220	11.4 (1.6)	11.5 (1.4)	0.021
Creatinine (mg/dL), median (IQR)	1.44 (1.19, 1.83)	1.73 (1.51, 2.23)	0.004	0.081	1.9 (1.4)	1.8 (0.9)	0.056
eGFR, mean (SD)	34.33 (13.50)	28.73 (11.06)	0.025	0.454	32.9 (11.7)	32.9 (13.9)	0.003
logistic_Euroscore, median (IQR)	11.68 (8.12, 17.16)	11.87 (7.97, 18.15)	0.897	0.027	13.7 (6.8)	13.6 (6.8)	0.012

BMI = body mass index; CABG = coronary artery bypass grafting; CAD = coronary artery disease; COPD = chronic obstructive pulmonary disease; CVA = cerebrovascular accident; eGFR = estimated glomerular filtration rate; MI = myocardial infarction; PAD = peripheral arterial disease.

**Table 2:** Baseline echocardiographic parameters in standard practice vs. the zero-contrast arm.

	Standard (*n* = 63)	Zero-Contrast (*n* = 44)	*p*	SMD	Standard (*n* = 63)	Zero-Contrast (*n* = 44)	Weighted SMD
EF, median (IQR)	55.00 (50.00, 55.00)	55.00 (50.00, 55.00)	0.590	0.171	53.3 (7.7)	53.8 (5.7)	0.060
Mean gradient, median (IQR)	50.00 (41.50, 57.50)	50.00 (41.75, 60.00)	0.807	0.022			
AVA, median (IQR)	0.70 (0.60, 0.80)	0.80 (0.70, 0.80)	0.216	0.087			
PAPs median (IQR)	30.00 (23.00, 45.00)	40.00 (30.00, 45.00)	0.063	0.321			
MR *n* (%)			0.156	0.476			
0	14 (22.2)	4 (9.1)					
1	28 (44.4)	28 (63.6)					
2	15 (23.8)	10 (22.7)					
3	6 (9.5)	2 (4.5)					
AR *n* (%)			0.727	0.231			
0	25 (39.7)	14 (31.8)					
1	20 (31.7)	18 (40.9)					
2	15 (23.8)	9 (20.5)					
3	3 (4.8)	3 (6.8)					

AR = aortic regurgitation; AVA = aortic valve area; EF = ejection fraction; MR = mitral regurgitation; PAPs = systolic pulmonary artery pressure.

**Table 3:** Main procedural data in the two groups of study.

	Standard (*n* = 63)	Contrast Zero (*n* = 44)	*p*	SMD
Pre-dilatation, *n* (%)	23 (36.5)	20 (45.5)	0.424	0.183
Post-dilatation, *n* (%)	14 (22.2)	16 (36.4)	0.129	0.315
Device implanted, *n* (%)			0.353	0.786
Evolut (Medtronic)	52 (82.5)	38 (86.3)		
Acurate (Boston)	10 (15.8)	3 (6.8)		
Lotus (Boston)	0 (0.0)	1 (2.2)		
Portico (Abbott)	1 (1.6)	1 (2.3)		
Navitor (Abbott)	0 (0.0)	1 (2.3)		

**Table 4:** Clinical events at follow-up in the two groups and their weighted Cox proportional hazard model.

	Standard (*n* = 63)	Contrast Zero (*n* = 44)	HR (95% CI)	*p*
Death from any cause, *n* (%)	18 (28.6)	5 (11.4)	0.71 (0.21–2.42)	0.58
CV death, *n* (%)	3 (4.8)	1 (2.3)	0.51 (0.04–6.92)	0.61
AKI, *n* (%)	10 (15.9)	2 (4.5)	0.05 (0.005–0.52)	0.01
Hospital readmission, *n* (%)	7 (11.1)	1 (2.3)	0.05 (0.01–0.26)	<0.001

AKI = acute kidney injury; CV = cardiovascular.

## Data Availability

The data presented in this study are available upon request from the corresponding author. The data are not publicly available due to data protection directive 95/46/EC.
